# Ginger (*Zingiber Officinale*)-derived nanoparticles in *Schistosoma mansoni* infected mice: Hepatoprotective and enhancer of etiological treatment

**DOI:** 10.1371/journal.pntd.0009423

**Published:** 2021-05-20

**Authors:** Wegdan M. Abd El Wahab, Ayman A. El-Badry, Soheir S. Mahmoud, Yaser A. El-Badry, Mohamed A. El-Badry, Doaa A. Hamdy

**Affiliations:** 1 Department of Medical Parasitology, College of Medicine, Beni-Suef University, Beni-Suef, Egypt; 2 Department of Microbiology-Medical Parasitology Section, College of Medicine, Imam Abdulrahman Bin Faisal University, Dammam, Saudi Arabia; 3 Department of Parasitology, Theodor Bilharz Research Institute (TBRI), Giza, Egypt; 4 Department of Chemistry, Faculty of Science, Taif University, Khurma, Taif, Saudi Arabia; 5 Organic Chemistry Lab., Faculty of Specific Education, Ain Shams University, Abbasseya, Cairo, Egypt; 6 Research Institute of Medical Entomology, General Organization for Teaching Hospitals and Institutes (GOTHI), Giza, Egypt; Texas Biomedical Research institute, UNITED STATES

## Abstract

**Background:**

Nanotechnology has been manufactured from medicinal plants to develop safe, and effective antischistosmal alternatives to replace today’s therapies. The aim of the study is to evaluate the prophylactic effect of ginger-derived nanoparticles (GNPs), and the therapeutic effect of ginger aqueous extract, and GNPs on *Schistosoma mansoni* (*S*. *mansoni*) infected mice compared to praziquantel (PZQ), and mefloquine (MFQ).

**Methodology/principal findings:**

Eighty four mice, divided into nine different groups, were sacrificed at 6^th^, 8^th^, and 10^th^ week post-infection (PI), with assessment of parasitological, histopathological, and oxidative stress parameters, and scanning the worms by electron microscope. As a prophylactic drug, GNPs showed slight reduction in worm burden, egg density, and granuloma size and number. As a therapeutic drug, GNPs significantly reduced worm burden (59.9%), tissue egg load (64.9%), granuloma size, and number at 10^th^ week PI, and altered adult worm tegumental architecture, added to antioxidant effect. Interestingly, combination of GNPs with PZQ or MFQ gave almost similar or sometimes better curative effects as obtained with each drug separately. The highest therapeutic effect was obtained when ½ dose GNPs combined with ½ dose MFQ which achieved 100% reduction in both the total worm burden, and ova tissue density as early as the 6^th^ week PI, with absence of detected eggs or tissue granuloma, and preservation of liver architecture.

**Conclusions/significance:**

GNPs have a schistosomicidal, antioxidant, and hepatoprotective role. GNPs have a strong synergistic effect when combined with etiological treatments (PZQ or MFQ), and significantly reduced therapeutic doses by 50%, which may mitigate side effects and resistance to etiological drugs, a hypothesis requiring further research. We recommend extending this study to humans.

## Author summary

Schistosomiasis is one of the most important tropical diseases that yearly causes millions of morbidities and mortalities. Several researchers are interested in trying variable chemical and herbal drugs for treating the disease. This research introduced a trial for prophylaxis and treating schistosomiasis mansoni in mice using ginger-derived nanoparticles (GNPs). GNPs have antischistosmal, antioxidant and hepatoprotective features. The nano-formulation of ginger provides a safer therapeutic and restorative enhancer to etiological treatments. GNPs may reduce infection complications, reduce required therapeutic doses of *Schistosoma*-standard drugs, and may help mitigate the side effects of and resistance to standard drugs; hypotheses which require further studies.

## Introduction

Schistosomiasis, the second most devastating tropical disease after malaria, annually affects 600 million individuals in 74 sub-tropical and tropical regions [1].

Praziquantel (PZQ), the selected drug for treating all *Schistosoma* sp., has been verified to be inefficient to treat juvenile worms, stop parasite transmission, inhibit reinfection or moderate induced morbidity, in addition to the emergence of resistant strains with long term administration of the drug, especially in endemic areas [2,3]. Although the antimalarial drug, mefloquine (MFQ), exhibits potential in vitro and in vivo antischistosomal effects, there is controversy about its side effects [4]. Thus, there is a pressing need to develop new safe and effective drugs to combat the development of resistance, and decrease drawbacks of both drugs.

In the last few years, there are increasing efforts to develop new antischistosomal drugs, especially using herbal sources. Among the promising medicinal plants that have been verified as an alternative antischistosomal compound is ginger (*Zingiber officinale* [*Z*. *officinale*]). This perennial herb shows various therapeutic effects as an antiemetic, antithrombotic, antibacterial, antifungal, antidiabetic, antioxidant, and anticarcinogenic [5]. In addition, *Z*. *officinale* exerts significant anti-helminthic, anti-protozoal, and anti-leech activity, as well as molluscicidal and insecticidal properties [6–7]. Significantly, as an antischistosomal drug, ginger improves the granulomatous inflammation, the main pathological sequalae associated with *Schistosoma* infection [8].

The advent of nanotechnology as a precise, fast, and cost-effective therapeutics has received considerable attention for the diagnosis and treatment of various infectious agents [9]. Owing to their small size, which ranges from 1 to 100 nm, nanoparticles can penetrate the very small capillaries throughout the body causing better solubility, absorption and uptake. In addition, nanoparticles highly aggregate more than normal drugs in the targeted tissues, typically contributing to decreased systemic toxicity [10].

Various nanoparticles such as gold, silver, and selenium have shown effective schistosomicidal properties [10,11]. Interestingly, nanoparticles have also been isolated from edible plants including ginger. As a natural product, ginger-derived nanoparticles (GNPs) require less complicated nanotechnology synthesis process than polymer-based nanoparticles. Several studies have successfully developed GNPs to treat alcoholic liver, and inflammatory bowel disease with promising results [12,13].

The current study was performed to evaluate the anti-schistosomal effect of ginger aqueous extract, and GNPs on *S*. *mansoni* infected mice compared to the standard anti-schistosomal drug (PZQ) and anti-malarial drug (MFQ).

## Materials and methods

### Ethics statement

The experimental animals were bred under appropriate conditions in compliance with the international standards, and the study was approved from the ethical committee at the Schistosome Biological Supply Program, Animal House of Theodor Bilharz Research Institute, Egypt.

### Experimental design

A total of 84 mice were divided into nine groups [G6, G7 (7 mice/each), other groups (10 mice/each)]. The first (G1), and second (G2) group were the negative (non-infected, non-treated), and positive (infected, non-treated) control groups respectively. The third group (G3) was given 5 mg/kg/mouse GNPs as prophylactic dose before *Schistosoma* infection. Other groups were designed as follow: G4 (infected-treated with ginger extract), G5 (infected-treated with GNPs), G6 (infected-treated with PZQ), G7 (infected-treated with ½ dose GNPs + ½ dose PZQ), G8 (infected-treated with MFQ), and G9 (infected-treated with ½ dose GNPs + ½ dose MFQ).

### Infection of mice

Male Swiss albino mice with average weight of 18–20 ± 2 gm were provided from Schistosome Biological Supply Program at Theodor Bilharz Research Institute, Egypt, and bred under specified pathogen-free conditions. With the exception of G1, mice in the remaining groups were subcutaneously infected with 60 ± 10 Egyptian strain of *S*. *mansoni* cercariae shed from *Biomphalaria alexandrina* snail according to Liang *et al*. [14].

### Preparation and characterization of ginger-derived nanoparticles

**Fig 1:**
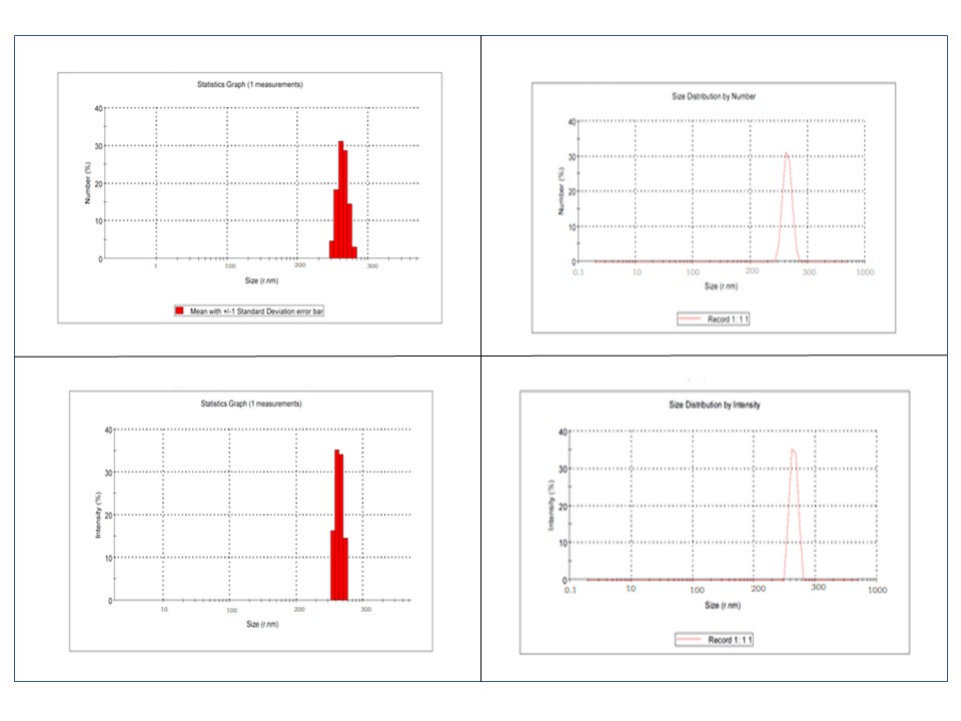
Identification and characterization of GNPs at pH 6.

GNPs were prepared from edible fresh ginger rhizome roots that were thoroughly washed three times with tap water. About 100 gm of washed ginger was blended with one litre of distilled water in a mixer for 10 minutes to make juice. The juice was filtered off and centrifuged at 3000×g for 20 minutes followed by 10,000×g for 40 minutes to eliminate large ginger fibers. The supernatant was decanted and subsequently ultra-centrifuged at 15,000×g for one hour two times. The formed pellet was collected as GNPs, suspended in phosphate buffered saline (PBS) via ultrasonic dispersion, and stored at −20°C until used. The obtained GNPs were identified using dynamic light scattering (DLS) to measure the distribution of particle size and zeta potentials [13], using Malvern instrument, United Kingdom (Zetasizer Ver. 6.32). GNPs pellets were obtained from ginger juice and were used without further purifications. The average GNPs size was about 238.3 nm and zeta potential value was about -13 mV at PH 6 (Fig 1).

### Drug preparation and dosage

Mice of G1 were orally injected by saline for 10 days (100 μl/mouse).

The aqueous extract of ginger was freshly prepared by suspending 30 gm of edible ginger rhizome roots in 60 ml of distilled water, followed by pressing out the homogenate through a cloth piece. The extract was orally administered to mice of G4 via stomach tube (500 mg/kg/mouse), three days/week, for 5 weeks starting from the 4^th^ week post-infection (PI) [15].

GNPs were vortexed in distilled water, and orally administered three days/week for 5 consecutive weeks starting from 4^th^ week PI at dose of 5 mg/kg/mouse (G5), and 2.5 mg/kg/mouse (G7 and G9). For prophylactic doses, mice of G3 were given prophylactic doses of GNPs (5 mg/kg/mouse) day after day one week before infection.

PZQ (Distocide, EIPICO, 10^th^ Ramadan, Egypt) was suspended in cremophore-El 2% (Sigma-Aldrich Chemical Co.). Mice were orally administered at 6^th^ week PI for G6 (1,000 mg/kg/mouse divided over two successive days), and G7 (500 mg/kg/mouse once) according to Muchirah *et al*. [16].

MFQ (Larium, F. Hoffmann-La Roche AG, Basel, Switzerland) was orally given as a fresh suspension in 3% ethanol, and 7% Tween-80 at a dose of 400 mg/Kg for G8, and 200 mg/Kg for G9 respectively at 3^rd^ week PI [17].

Mice from each group were sacrificed at 6^th^, 8^th^, and 10^th^ week PI by decapitation. At each time, liver, and intestinal tissues were collected for parasitological, and histopathological studies. At the end of the experiment, adult worms were collected, scanned under the scanning electron microscope (SEM), and parts of the liver tissues were homogenized for determination of oxidative stress markers.

### Parasitological studies

#### Worm recovery

Sacrificed mice were exposed to hepato-portomesentric perfusion technique to collect adult *S*. *mansoni*, detect sex (male/female/copula), determine worm burden, and then calculate the percentage of reduction of total worms [18].

#### Oogram count

Three intestinal samples (1 cm/each) were tested for eggs at different developmental stages. According to the criteria identified by Pellegrino *et al*. [19], the mean percentage of eggs at each stage/animal/group was calculated.

#### Egg count in tissues

Small pieces of hepatic and intestinal tissues were weighted, digested overnight in 5 ml KOH 5% solution, and three samples (each 50 μl) of the digested tissue were microscopically examined to determine the mean egg count [19]. Number of eggs/gram tissue, and the percentage of reduction of total ova/gram tissue was calculated according to Kloetzel [20].

### Pathological studies

From each group, a portion of liver tissue was immediately fixed in 10% formalin, dehydrated, prepared for paraffin sectioning, and stained using hematoxylin and eosin [21]. At the end of experiment, the mean diameter of granuloma (μm) for each group was calculated from five fields from different sections and different mice. Also, the mean number of granulomas, granuloma type (fibrous/ cellular/ fibrocellular), and egg state (intact/degenerated) were assessed.

### Assessment of oxidative stress markers

Liver tissues were perfused in PBS containing 0.16 mg/ml heparin (pH = 7.4) to eliminate red cells or blood clots. Then, liver tissues were homogenized in 5 ml cold buffer (1 mM EDTA, 1 ml/L Triton X-100, and 50 mM potassium phosphate)/gram tissue. The tissue homogenate was centrifuged for 15 min at 4000 rpm at 4°C, followed by collection of the supernatant and freezing at −80°C till assayed.

#### Determination of catalase (CAT) activity

CAT activity was spectrophotometrically measured according to Sinha [22]. Absorbance of sample was measured at 510 nm. CAT (U/gm) was calculated according to the manufacturer instructions (Biodiagnostics, Co.)

#### Determination of L-malondialdehyde (MDA) activity

MDA was calorimetrically determined following the method adapted by Esterbauer et al. [23], where serum lipid peroxidation was estimated by determination of thiobarbituric acid reactive substance (TBARs), a colored complex. Sample absorbance was read at 534 nm and expressed as μmol/gm following the manufacturer’s instructions (Biodiagnostics, Co.)

#### Scanning electron microscope (SEM) examination

To estimate the extent of surface damage, adult worms were fixed in glutaraldehyde buffer solution 4% in sodium cacodylate buffer for a range of 2 to 24 h. Then, they were washed in osmic acid buffer, followed by distilled water, rising concentrations of alcohol (30–90%), and finally in 100% alcohol. Adult worms were mounted on stubs, coated with gold and carbon, then measured with Joel-1200 EX2 SEM at scanning microscope photographing unit, Theodor Bilharz Research Institute, Egypt [8].

### Statistical analysis

Collected study data were tabulated, and statistically analyzed using the software, Statistical Package for Social Sciences (SPSS) version 26. Quantitative date was expressed as mean ± SD. ANOVA test was used to assess differences among groups for all parameters, including total worm burden, egg developmental stages, number of egg/gm tissue, granuloma diameter, number, and type, egg state, and oxidative stress markers values. Post-hoc test was used for pairwise comparison of all paired groups (Tukey high significant degree). *P* value was considered significant if it was equal or less than 0.05.

## Results

The worm burden was assessed for each experimental group at 6^th^, 8^th^, and 10^th^ weeks PI. The complete reduction in the total worm burden (100%) was at the 6^th^ week PI in G9 treated with ½ dose GNPs and ½ dose MFQ, and at 10^th^ week PI in G6 and G8 treated with PZQ and MFQ respectively with statistical significance of the results (*P*<0.001) compared to infected non treated control group (G2). A gradual worm reduction of statistical significance occurred in G4 and G5 treated with ginger aqueous extract and GNPs, respectively with the best results were obtained at 10^th^ week PI (G4: 44.6%, G5: 59.9%). However, slight reduction in the worm burden (27.7%) of statistical significance occurred in G3 at 10^th^ week PI after administration of GNPs as a prophylactic dose before *Schistosoma* infection (Table 1).

**Table 1:** The mean worm burden in *S*. *mansoni* infected mice sacrificed at 6^th^, 8^th^, 10^th^ weeks PI in different experimental groups.

Animal group	6 weeks PI					8 weeks PI					10 weeks PI				
	Mean worm burden ± SD			Total worm burden	% reduction of total worm	Mean worm burden ± SD			Total worm burden	% reduction of total worm	Mean worm burden ± SD			Total worm burden	% reduction of total worm
	Male	Female	Couples			Male	Female	Couples			Male	Female	Couples		
**G2**	3.8 ± 0.51	1.02 ± 0.31	6.4 ± 0.76	17.62 ± 2.25	-----	3.2 ± 0.25	1.02± 0. 13	7.02 ± 2.26	18. 26 ±2.23	-----	3.04 ± 0.22	0	7.12± 1.20	17. 28± 2.10	-----
**G3**	3.55 ± 0.58	1.2 ± 0.31	4.39 ± 0.42	13.53 ± 1.87	23.2	3.15 ± 0.58	1.02± 0.31	5.17 ± 0.43	14.51 ± 1.22	20.5	3.45 ± 0.38	0.7 ± 0.01	4.17 ± 0.53	12.49 ± 1.12*	27.7
**G4**	3.83 ± 0.49	1.01± 0.24	3.80 ± 0.08	12.44 ± 1.65*	26.4	2.58 ± 0.23	0	4.72 ± 0.46	12.02 ± 0.37*	34.2	2.73 ± 0.24	0	4.42 ± 0.15	9.57 ± 0.42*	44.6
**G5**	2.57 ± 0.96	0	4.40 ± 0.30	11.37± 2.54*	35.5	2.63 ± 0.29	0	4.12 ± 0.18	10.87 ± 0.45*	40.5	2.38 ± 0.23	0	5.27 ± 0.56	6.92 ± 0.47*	59.9
**G6**	----	----	----	----	----	1.02± 0.02	0	0	1.02± 0.02*	94.4	0	0	0	0*	100
**G7**	----	----	----	----	----	1.02 ± 0.06	0	1.04 ± 0.03	3. 1± 0.05*	83	1 ± 0.02	0	1 ± 0.02	2.53±0.02*	85.5
**G8**	1.42 ± 1.27	0	4.71 ± 3.49	4.71 ± 3.49*	80.2	1.0± 0.02	0	0	0.80 ± 0.58*	94	0	0	0	0*	100
**G9**	0	0	0	0*	100	0	0	0	0*	100	0	0	0	0*	100

NB: G6 and G7 were not included at 6^th^ weeks PI as PZQ dose was administered at the same period.

* P value < 0.05. Values are given as mean ±SD for 3 mice in each group at 6^th^, 8^th^ weeks PI and 4 mice in each group at 10^th^ weeks PI. G2; infected non-treated, G3; prophylaxis by GNPs then infected, G4; infected-treated with ginger extract, G5; infected-treated with GNPs, G6; infected-treated with PZQ, G7; infected-treated with 1/2 dose GNPs + 1/2 dose PZQ, G8; infected-treated with MFQ, and G9; infected-treated with 1/2 dose GNPs + 1/2 dose MFQ.

Concerning oogram pattern, G9 treated with ½ dose GNPs and ½ dose MFQ showed a complete reduction (100%) of hepatic and intestinal tissue egg density at 6^th^ week PI, followed by G6, treated with PZQ (100%), and G8 treated with MFQ (98%) at 10^th^ week PI, with statistical significance (*P*<0.001) in relation to infected non treated control group (G2). G4 treated with ginger extract and G5, treated with GNPs, achieved best results of 10^th^ week PI (G4: 42.2%, G5: 64.9%), with statistical significance, which was mainly associated with a gradual increase in the total percentage of dead ova count over time. G3 showed significant decrease in the hepatic tissue egg load compared to G2, with low reduction of total ova/gram tissue (10.2%) at 6^th^ week PI (Table 2).

**Table 2:** The percentage of egg developmental stages and number of ova/gram tissue in mice sacrificed at 6^th^, 8^th^, 10^th^ weeks PI in different experimental groups.

Animal group	6 weeks PI						8 weeks PI						10 weeks PI					
	% egg developmental stages			Number of ova/gm tissue		% reduction of total ova/ gm tissue	% egg developmental stages			Number of ova/gm tissue		% reduction of total ova/ gm tissue	% egg developmental stages			Number of ova/gm tissue		% reduction of total ova/gm tissue
	immature ova	Mature ova	Dead ova	Liver	Intestine		immature ova	Mature ova	Dead ova	Liver	Intestine		immature ova	Mature ova	Dead ova	Liver	Intestine	
**G2**	50	45	5	8217.08 ± 311.79	9263.39 ± 633.85	-----	50	40	10	9217.08 ± 378.79	10263.39 ± 933.89	-----	45	37	18	9217.08 ± 378.79	10263.39 ± 933.89	-----
**G3**	50	44	6	7269.41 ±215.46*	8427.42 ±534.22	10.2	42	50	8	8769.66± 325.46	9427.58 ±354. 2	6.6	42	48	10	9496.26 ± 235.42	9542. 85 ± 345.19	2.3
**G4**	40	52	8	6882.75± 521.44*	8633.45 ±357.48	11.2	43	45	12	5500 ± 148.25*	6250.76± 358.24*	39.7	43	40	17	5172.41 ±24.24*	6082 ± 57.38*	42.2
**G5**	42	45	13	6871.28 ±537.25*	7365.53 ±482.17*	18.6	35	50	15	2272 ± 21.24*	5882 ± 37.38*	58.1	35	45	20	3970± 158.27*	2870.76 ±318.24*	64.9
**G6**	----	---	-----	-----	-----	-----	0	5	95	833 ± 23.07*	384.61 ± 24.22*	93.8	0	2	98	173.23 ± 21.00*	214.41 ±14.20*	98
**G7**	----	----	----	-----	-----	-----	20	15	65	1666.75 ±357.15*	2465.23 ±248.27*	78.8	10	15	75	1246.75 ±375.12*	2645.23 ±241.24*	80
**G8**	10	35	55	14.562 ± 7.928	3.082 ±742*	63.1	0*	0*	0*	568.45 + 34.02*	145.03 + 27.33*	96.3	0*	0*	0*	0*	0*	100
**G9**	0*	0*	0*	0*	0*	100	0*	0*	0*	0*	0*	100	0*	0*	0*	0*	0*	100

NB: G6 and G7 were not included at 6^th^ weeks PI as PZQ dose was administered at the same period.

* P value < 0.05. Values are given as mean ±SD for 3 mice in each group at 6^th^, 8^th^ weeks PI and 4 mice in each group at 10^th^ weeks PI. G2; infected non-treated, G3; prophylaxis by GNPs then infected, G4; infected-treated with ginger extract, G5; infected-treated with GNPs, G6; infected-treated with PZQ, G7; infected-treated with 1/2 dose GNPs + 1/2 dose PZQ, G8; infected-treated with MFQ, and G9; infected-treated with 1/2 dose GNPs + 1/2 dose MFQ.

**Fig 2:**
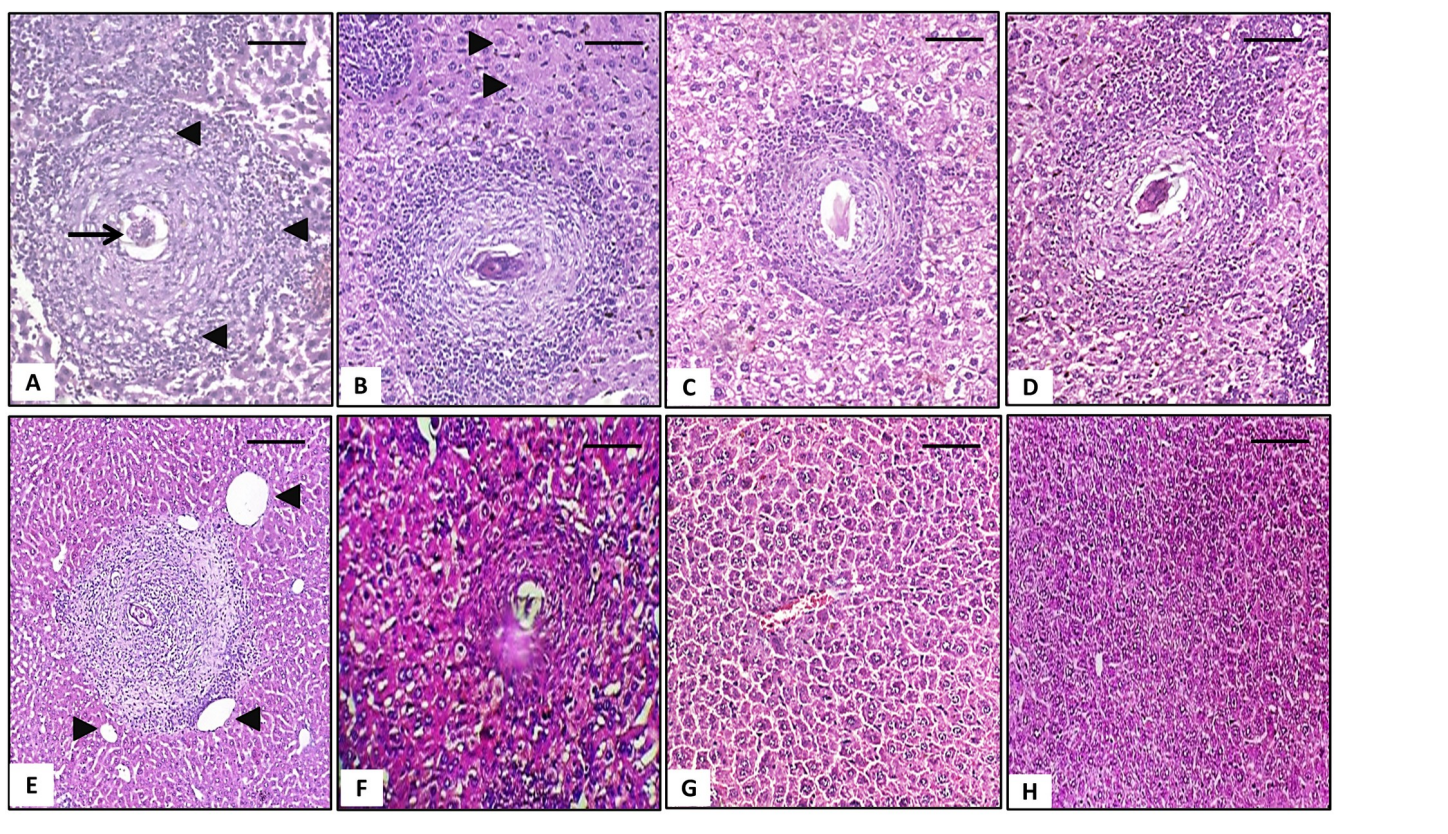
Histopathological liver sections of different *S*. *mansoni*-infected groups stained with hematoxylin and eosin (×100). **A** Infected non-treated group showing a large cellular granuloma, composed of inflammatory cellular infiltrate (black arrow head) surrounding central trapped ova (black arrow), **B** Prophylaxis group showing large granuloma around trapped fresh egg and some vacuolated hepatocytes with cloudy changes (black arrow head), **C** Ginger treated group showing reduced granuloma size with vacuolated cytoplasm, **D** GNPs treated group showing significant smaller granuloma size; with less cellular infiltration, **E** PZQ treated group showing remarkably reduced granuloma size with less inflammatory cellular infiltration surrounded by proliferating fibroblast, normal hepatocytes architecture, congested central vein (arrow head) and moderate congestion of hepatic sinusoids, **F** PZQ/ GNPs treated group showing decreased size of the granuloma with dilated liver sinusoids, **G and H** MFQ and MFQ/GNPs treated group showing nearly-preserved liver architecture with intact hepatocytes without any detected eggs or granuloma formation. *Bar = 100 μm*.

Histopathological examination of hepatic tissues of positive control group (G2) showed chronic granulomatous inflammation marked by numerous granulomas and inflammatory cellular infiltrations composed mainly of lymphocytes, epitheloid cells, and eosinophils. The normal hepatic, and lobular structure was in turn disrupted by the multiple granulomas. Hypereosinophilia, vacuolar degeneration, and necrotic foci appeared in the hepatocytes. Portal venules, and central veins were dilated with infiltration of inflammatory cells in portal spaces. Examination of liver sections from different treated groups revealed a significant decrease in the granulomas size, and number with concentric fibrosis around the trapped *Schistosoma* ova and many fibroblasts. Dilated liver sinusoids with lymphocytic infiltration were revealed. In G8 treated with MFQ, and G9 treated with ½ dose GNPs and ½ dose MFQ, the organization of liver architecture was almost restored and most hepatocytes appeared to be normal (Fig 2).

The mean granuloma diameter, number of granulomas, types of granuloma, and state of *S*. *mansoni* eggs in all experimental groups were summarized in Table (3). There was an absence of granuloma formation in G8, treated with MFQ, and G9 treated with ½ dose GNPs and ½ dose MFQ. In G7 treated with ½ dose GNPs and ½ dose PZQ, the mean diameter and number of hepatic granulomatous inflammation was significantly reduced, and the percentage of degenerated eggs was increased concomitant with an obvious decrease in cellular granuloma type, replaced by fibro-cellular granuloma.

**Table 3:** The mean granuloma diameter and number in five fields (×100), types of granuloma, and state of *S*. *mansoni* eggs in mice sacrificed 10^th^ weeks PI in different experimental groups.

Animal groups	Granuloma diameter (μm)	Granuloma number in five fields (×100) mean ± SD	% Granuloma type			% *S*. *mansoni* eggs state	
			Cellular	Fibro cellular	Fibrous	Intact	Degenerated
**G2**	368±25.23	12.67±1.37	42	58	0	84	16
**G3**	349±41.53	9.75±1.92	45	55	0	80	20
**G4**	334±37.21	11.67±1.63	40	60	0	73	27
**G5**	315.42±18.3*	8.34+1.7*	48	52	0	60*	40*
**G6**	265.3±28.51*	7.5±2.51*	45	55	0	57*	43*
**G7**	246±21.24*	4.83±0.7*	35	65	0	40*	60*
**G8**	0*	0*	0*	0*	0*	0*	0*
**G9**	0*	0*	0*	0*	0*	0*	0*

* P value < 0.05. Values are given as mean ±SD for 4 mice in each group. G2; infected non-treated, G3; prophylaxis by GNPs then infected, G4; infected-treated with ginger extract, G5; infected-treated with GNPs, G6; infected-treated with PZQ, G7; infected-treated with 1/2 dose GNPs + 1/2 dose PZQ, G8; infected-treated with MFQ, and G9; infected-treated with 1/2 dose GNPs + 1/2 dose MFQ.

**Fig 3:**
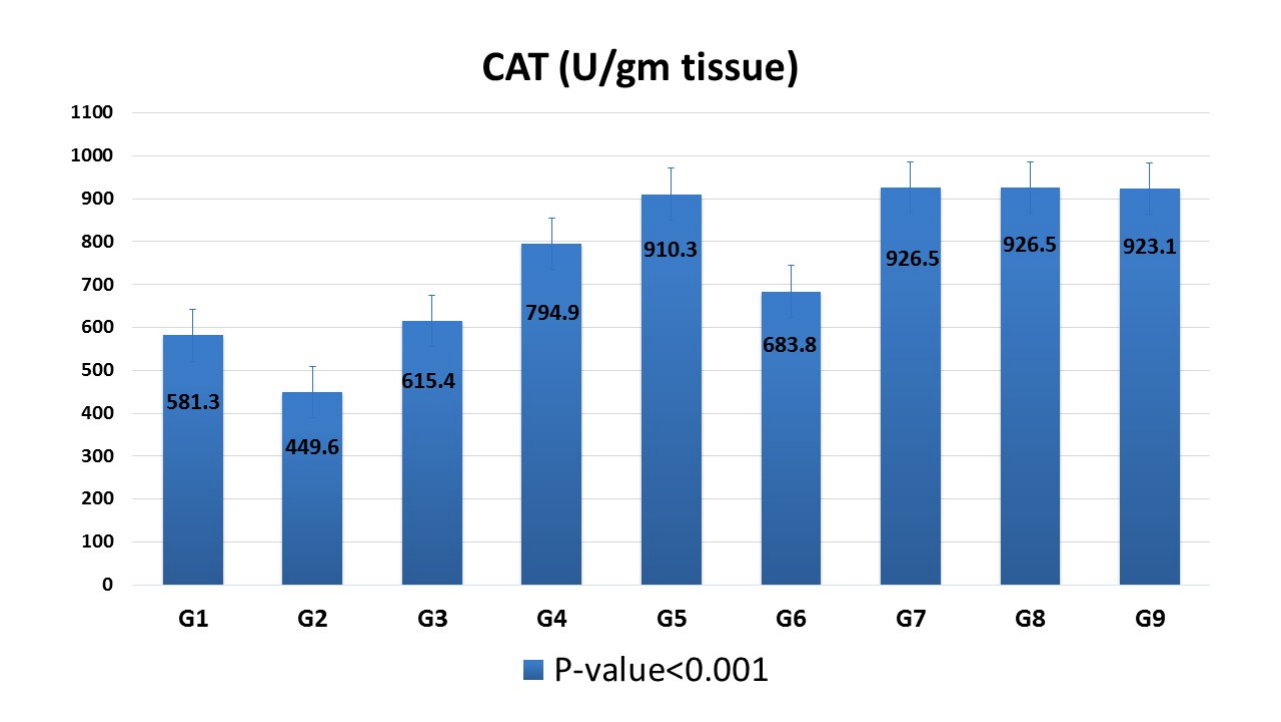
Effect of different treatment regimens included in the study on liver CAT level of *S*. *mansoni* infected mice groups sacrificed at 10^th^ week PI. Values are given as mean ±SD for 4 mice in each group. Values show high significant difference (*P* < 0.001).

Data represented in Fig 3, show the significant decrease in mean CAT level (*P* <0.05) in liver tissues of G2 infected non-treated group (449.6 U/gm), as compared to G1 non-infected non-treated group (581.3 U/gm). However, after administration of different treatment regimens included in the study, the level of CAT increased with high statistical significance (*P* <0.001) in comparison to the corresponding G2 infected non-treated group.

**Fig 4:**
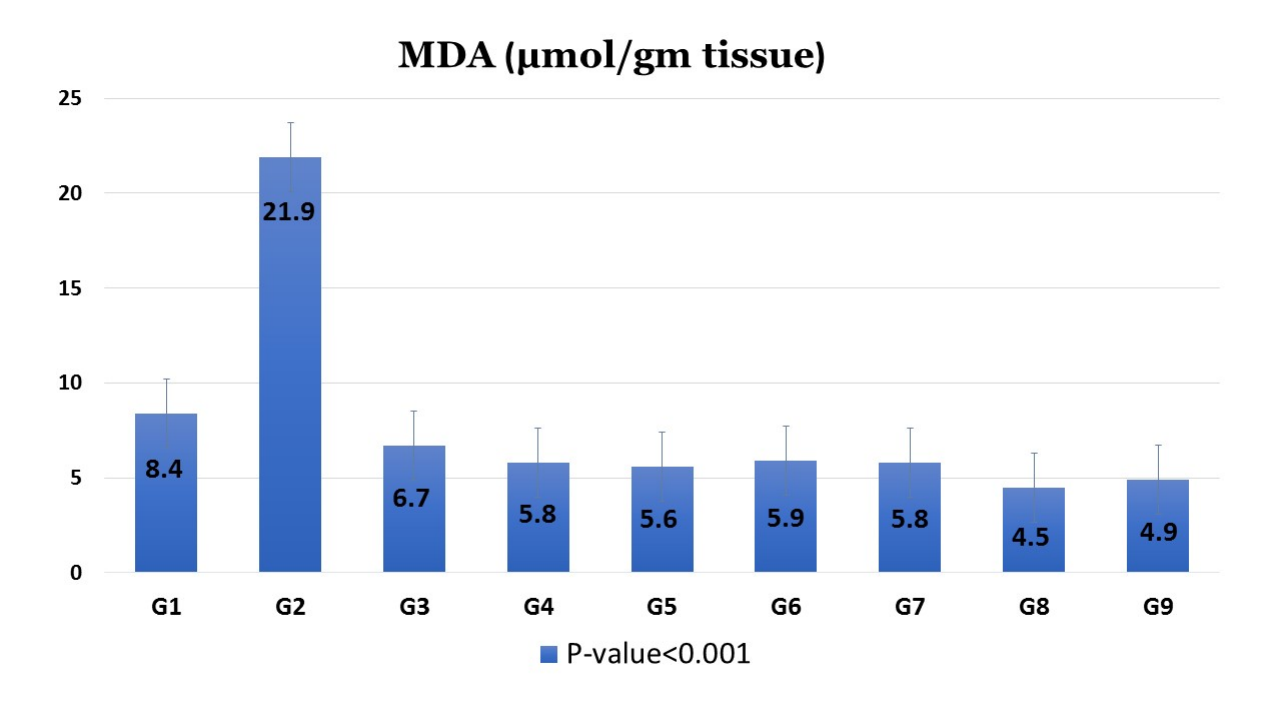
Effect of different treatment regimens included in the study on liver MDA level of *S*. *mansoni* infected mice groups sacrificed at 10^th^ week PI. Values are given as mean ±SD for 4 mice in each group. Values show high significant difference (*P* < 0.001).

As illustrated in Fig 4, the obtained results revealed significant (*P* < 0.05) increase in the mean hepatic MDA level after *S*. *mansoni* infection in G2 (21.9 μmol/gm) as compared to the non-infected, non-treated control group G1 (8.4 μmol/gm). However, after administration of different treatment regimens included in the study, the level of MDA decreased with high statistical significance (*P* <0.001) in relation to the corresponding G2, infected non-treated group.

**Fig 5:**
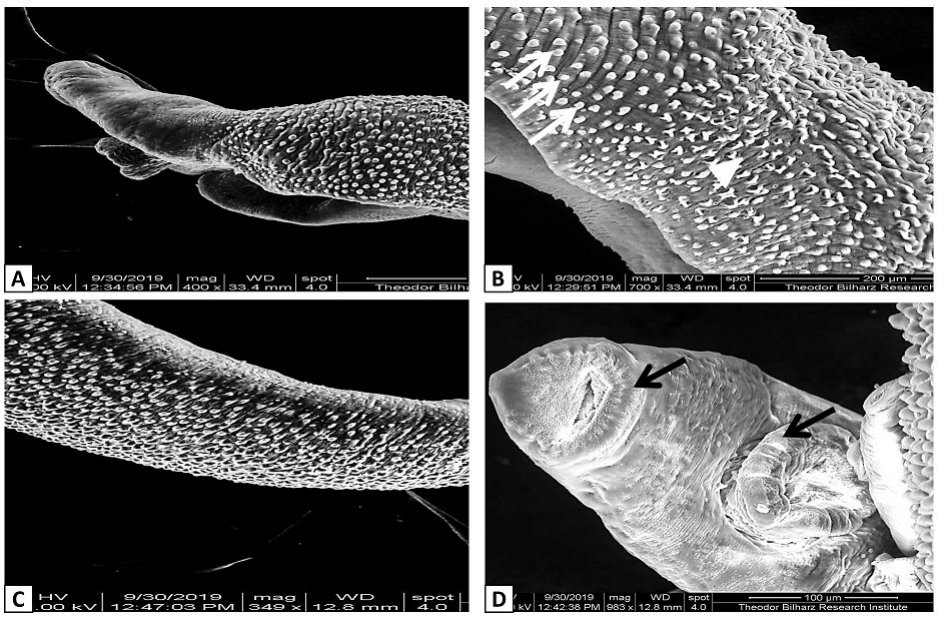
**Scanning electron micrograph of dorsal surface of adult male *S*. *mansoni* recovered from** **A and B** Infected non-treated mice (G2) showing numerous tubercles bearing directed spines (white arrows) and intertubercular ridges (white arrow head); **C and D** Prophylaxis group (G3) showing mild distortion of the tubercles bearing short and blunt spines on the dorsal surface with prominent oral and ventral suckers (black arrows).

**Fig 6:**
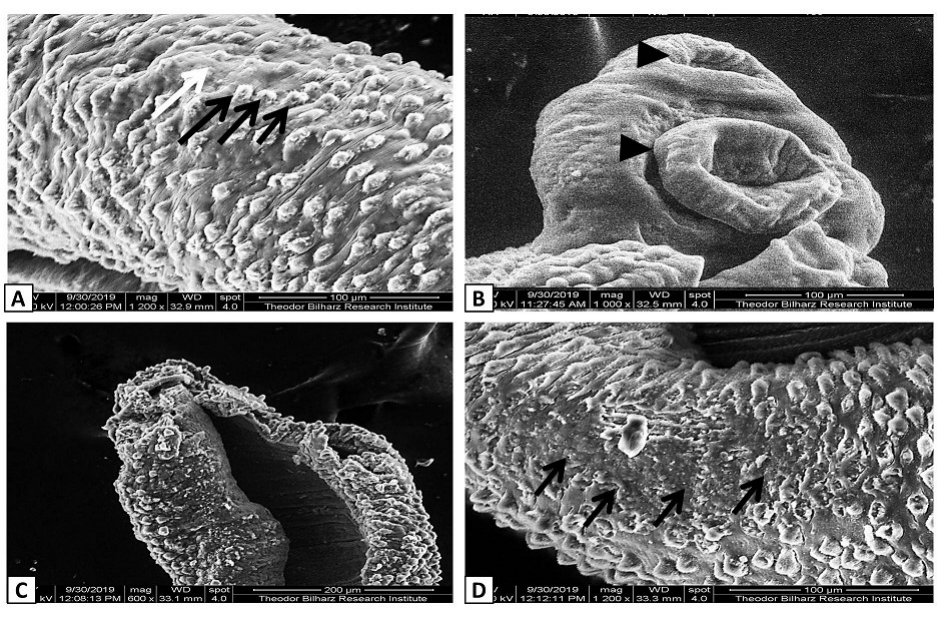
**Scanning electron micrograph of dorsal surface of *S*. *mansoni* male worms recovered from A and B** Ginger treated mice (G4) showing moderate distortion of the tubercles with short and blunt spines (black arrows), loss of intertubercular ridges (white arrow), and softening and flattening of oral and ventral suckers (black arrow head); **C and D** GNPs-treated mice (G5) showing extreme deformity and abnormal surface architecture in the form of flattened tubercles that lost their spines and appeared torn at the tip (black arrows).

**Fig 7:**
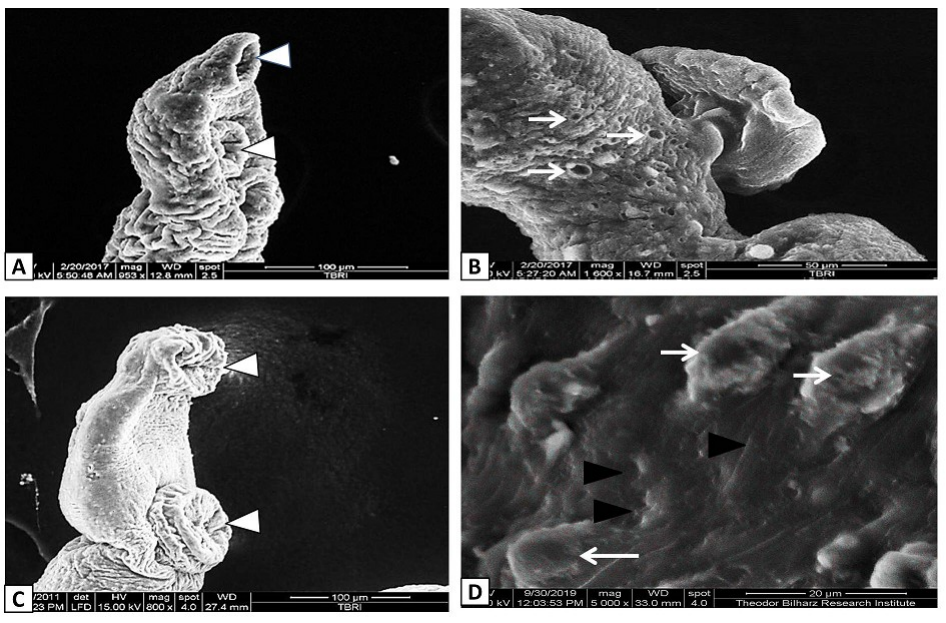
**Scanning electron micrograph of dorsal surface of *S*. *mansoni* male worms recovered from A and B** PZQ-treated mice (G6) showing deformed and swollen oral and ventral suckers (white arrow head), added to extreme deformity, more swelling, poring (white arrows) and furrows on the tegmental surface with flattening of tubercles and fall of spines; **C and D** PZQ with GNPs- treated mice (G7) showing abnormal swelling and flattening of oral and ventral suckers (white arrow head) in addition to blunt flattened tubercles and loss of spines (white arrow), with extensive erosion and cracking (black arrow head).

SEM examination showed that, the dorsal tegumental surface of male *S*. *mansoni* worms in G2 infected non-treated group had large multiple tubercles with numerous intact spines. While, male *Schistosoma* worms recovered from other different experimental groups showed wide variable alterations in tegumental normal architecture. Flattened and distorted oral suckers, partial or complete loss of spines, and extensive erosions around tubercles were observed in some worms (Figs 5–7).

## Discussion

PZQ and MFQ are two drugs that show efficient antischistosomal effect, but with some degree of conflict about their limitations, and side effects [4]. Researchers are motivated to develop natural, safe, and effective alternatives to overcome drawbacks of both drugs.

Based on findings of the study, ginger aqueous extract significantly affects egg development (inducing more degenerated eggs), worm fecundity (decreasing egg deposition), and is lethal to the adult worms leading to a decrease in worm burden. In addition, ginger reduces granuloma number and size which is habitually a huge sequel of schistosomiasis, and ends fatally.

These results agree with Hassan *et al*. [8], Mostafa *et al*. [15], and Al-Sharkawi *et al*. [24]. In contrast, Sanderson *et al*. [25] assessed the activity of ginger ethanolic extract on *S*. *mansoni* adult worms and didn’t find any significant decrease in egg counts in liver or worm burden as compared to untreated control groups. This may be due to variations in dosage, and type of extract used in the experiment.

The study examined the possible prophylactic effect of GNPs on *Schistosoma* infection for the first time; however, GNPs couldn’t prevent *Schistosoma* infection. There was slight decrease in the worm burden (27.7%) at 10^th^ week PI, minimal reduction of total ova/gram tissue (10.2%) at the 6^th^ week PI, and reduction in granuloma size and number without statistical significance. This prophylactic effect may be enhanced by another route of administration, higher or more repeated doses, or combination with other synergetic drugs.

In the present study, administration of GNPs achieved significant reduction in worm burden, and the hepatic and intestinal egg load, with the best results obtained at 10^th^ week PI. Combined ½ dose GNPs and ½ dose PZQ, had nearly the same effect as PZQ alone, which enhances the synergistic role of GNPs. After GNPs administration, liver granuloma diameter and number significantly decreased, and the type of granuloma became more fibro-cellular. There was a significant increase in the number of degenerated ova with the maximum effect occurring when ½ the dose of GNPs combined with ½ the dose of PZQ that was better than results of PZQ alone.

In the current study, the highest and best results of GNPs antischistosomal effect were obtained early in the 6^th^ week PI, when ½ the dose of GNPs was combined with ½ the dose of MFQ, there was 100% reduction of total worm burden, 100% reduction of egg density in liver and intestinal tissues, and no egg detection or granuloma formation. The normal organization of liver architecture was nearly restored, and most hepatocytes appeared normal. These results gave better significant effect than MFQ alone (*P* < 0.001).

Muscular contraction, mechanical destruction, and metabolic disorders of treated worms by antischistosomal drugs might be the possible cause of worm’s death. The decrease of egg tissue load in treated mice might be due to the decrease in the burden of total worms after treatment, the low female productivity, and the host’s tissue reaction that actively destructed the few produced eggs [26].

Great promise for treatment enhancement may be achieved by targeting and designing variable nanoparticle dependent drug carriers. Several carriers have already been prepared at certain pH, to resist digestive enzymes, and/or need specific bacterial cleavage for activation. Fabrication of medical nanoparticles using plants as nano-factories might introduce a new approach in the medical field [13], and this was an important goal of the present research.

The average GNPs size (238.3 nm) in the present study and zeta potential value (-13 mV at PH 6) are to a large extent in agreement with Zhang et al [13]. Nano-formulation of GNPs resulted in almost 13-fold smaller particle size compared to Zhuang et al. [12] and Norhidayah et al. [27]. The number and intensity of particle size distribution exhibited a uniform distribution and polydispersity reduction of GNPs (Fig 1).

In the current study, GNPs were administered via the oral route which is more advantageous than other therapeutic routes. Oral therapy achieves the purpose of carrying GNPs to the mesenteric venous plexus, the main habitat of *S*. *mansoni*. The obtained results are consistent with prior studies, which reported that oral administration of nanoparticles showed transitional stability down to the colon. Moreover, the oral route of administration didn’t trigger any systemic or local side effects [13,28].

Another novelty in the present study, is the modification in GNPs preparation, which facilitated their dissolving in distilled water. This increases the bioactivity of the nanoparticles, ensures their complete absorption as good as venous administration, and raises their safety.

The promising effect of ginger and GNPs on granulomatous reaction is more or less similar to other researchers using similar treatments [15]. This may be attributed to Th1 and Th2 lymphocytes suppression with subsequent decrease in related cytokines, which play a major role in the formation of granuloma as mentioned by Wynn and Cheever [29]. Restoring normal hepatic organization and hepatocyte appearance in ginger extract and GNPs treated groups may be due to the potent antioxidant effect of ginger, and its ability to scavenge free radicals [30]. Schistosomiasis is associated with a disturbance in the cellular antioxidant mechanism, and liberation of free radicals. In an established infection, the host immune system generates oxidative stress and liberates oxygen-derived free radicals as a non-specific initial defense to counteract the antioxidant defense mechanism of the parasite [31].

CAT is a vital ingredient of the antioxidant defense mechanism. It protects against oxidative damage by removal of toxic free radicals, and keeps homeostasis [32,33]. The present study revealed that CAT activity of the liver tissue of the positive control mice group (G2) decreased significantly as compared to non-infected, non-treated control group (G1). Rizk *et al*. [34] illustrated that CAT activity had been reduced as it was utilized in scavenging overload of the free radicals that are frequently liberated in schistosomiasis. This may be attributed to the increased oxygen metabolites in schistosomiasis leading to a decrease in the host antioxidant activity.

Treatment with ginger extract and GNPs in the different experimental groups increased the activity of the hepatic tissue CAT significantly as compared to the infected non treated mice group (G2) (*P* < 0.001), which is in agreement with many researches, which have also shown that ginger enhances the level of antioxidant enzyme [8,35,36,37]

In the present study, the level of MDA, lipid peroxidation end product, was notably elevated in the liver of the positive control mice group (G2). This elevated MDA level indicates strong lipid peroxidation with a failure of the host antioxidant defense mechanisms to prevent excessive free radical formation and tissue damage [38,39]. Additionally, reduction of the antioxidant capability of the liver in infection causes more liberation of lipid peroxides which have a great effect in the pathology of schistosomiasis [40].

Treatment with ginger extract and GNPs in the different experimental groups reduces the MDA level significantly (*P* < 0.001), which may indicate that the hepatoprotection is due to its antioxidant activity. This finding is in agreement with El-Derbawy *et al*. [37]. The antioxidant effect of ginger and GNPs may be attributed to the inhibition of lipid peroxidation and/or stimulation of the antioxidant enzymes. On the other hand, Baliga *et al*. [41] referred the antioxidant activity of ginger to the ability of the main constituent of ginger, zingerone, to scavenge O_2_ and OH radicals.

The results concerning GNPs were unique and in spite of scarcity of similar studies, the results partially agreed with El-Derbawy *et al*. [37]. who showed that ginger loaded on chitosan nanoparticles decreased the produced oxidative damage of liver cells, and have antischistosomal activities. Previous studies have successfully developed GNPs to treat alcoholic liver [12], and inflammatory bowel disease [13] with promising results.

Since *Schistosomes* tegument is an important target for antischistosomal drugs, morphological changes in the worm surface topography were used by previous researchers for evaluation of activities of some antischistosomal drugs [15]. The alterations caused by antischistosomal drugs on adult worm teguments were more noticeable in males than females, as most of the female worms are enclosed in the male gynaecophoric canal, rendering it away from the direct host’s defense mechanism [42]. Accordingly, the adult male worms were selected to study the anti-tegumental effects of ginger extract, and GNPs.

The morphological alterations in ginger extract and GNP treated mice groups were to some extent similar to the tegumental alterations obtained by Mostafa et al. [15]. Such damage of the suckers might lead to loss of the worm’s ability to firmly adhere to the venules wall, making absorption of blood nutrients more difficult. Deterioration of the tegument a lengthwise the whole worm would impair its normal function and damage its defense mechanism as well, hence it could be readily overcome by the host’s immune response [43]. Similar to the mentioned application of ginger extract in previous literature [6], GNPs can be successfully employed for treatment of variable neglected tropical parasitic diseases, including toxocariasis, aniskiasis, hydatidosis, african trypanosomiasis, giardiasis, blastocystiasis, and toxoplasmasmosis.

Interestingly, ginger alone or combined with other plants enriched with vitamin C was effectively used as a prophylactic or therapeutic home remedies in the management of COVID-19 [44]. Also, ginger improved clinical symptom recovery rate (tiredness, dry cough, fever), and paraclinical features (C-reactive protein, lymphocytopenia, and thrombocytopenia,) of patients with severe acute respiratory syndrome due to COVID-19 within seven days [45].

Limitations of the present study: the potential effects of GNPs combined with etiological *Schistosoma* treatment on reduction of drug resistance and side effects, as well as the prevention of complications of chronic schistosomiasis was not evaluated and would necessitate further studies.

## Conclusion

Based on our findings, GNPs have an antischistosomal effect, ameliorate the host oxidative stress and have a hepatoprotective effect. When combined with the regular etiological treatment, both PZQ and MFQ, GNPs significantly reduced therapeutic doses by 50%, almost similar to the administration of the full dose of each drug, suggesting its synergistic effect. In addition to its effect on *S*. *mansoni* worms, GNPs restored hepatocytes and maintained normal hepatic organization, which may contribute towards reducing the complications of chronic schistosomiasis. Subsequent research on the role of GNPs in the reduction of *Schistosoma* complications, as well as the mitigation of side effects and resistance to etiological therapeutic drugs are still needed. We recommend extending this study to human trials to assess the therapeutic role of GNPs for schistosomiasis.
